# Candidate Gene Approach Identifies Multiple Genes and Signaling Pathways Downstream of Tbx4 in the Developing Allantois

**DOI:** 10.1371/journal.pone.0043581

**Published:** 2012-08-28

**Authors:** Ripla Arora, Chelsea M. del Alcazar, Edward E. Morrisey, L. A. Naiche, Virginia E. Papaioannou

**Affiliations:** 1 Department of Genetics and Development, Columbia University Medical Center, New York, New York, United States of America; 2 Department of Medicine and Cell and Developmental Biology, University of Pennsylvania, Philadelphia, Pennsylvania, United States of America; 3 Cancer and Developmental Biology Laboratory, National Cancer Institute, Frederick, Maryland, United States of America; Brigham and Women’s Hospital, United States of America

## Abstract

Loss of *Tbx4* results in absence of chorio-allantoic fusion and failure of formation of the primary vascular plexus of the allantois leading to embryonic death at E10.5. We reviewed the literature for genes implicated in chorio-allantoic fusion, cavitation and vascular plexus formation, processes affected in *Tbx4* mutant allantoises. Using this candidate gene approach, we identified a number of genes downstream of *Tbx4* in the allantois including extracellular matrix molecules *Vcan, Has2*, and *Itgα5*, transcription factors *Snai1* and *Twist*, and signaling molecules *Bmp2*, *Bmp7, Notch2, Jag1* and *Wnt2*. In addition, we show that the canonical Wnt signaling pathway contributes to the vessel-forming potential of the allantois. *Ex vivo*, the *Tbx4* mutant phenotype can be rescued using agonists of the Wnt signaling pathway and, in wildtype allantoises, an inhibitor of the canonical Wnt signaling pathway disrupts vascular plexus formation. *In vivo*, *Tbx4* and *Wnt2* double heterozygous placentas show decreased vasculature suggesting interactions between *Tbx4* and the canonical Wnt signaling pathway in the process of allantois-derived blood vessel formation.

## Introduction

The chorio-allantoic placenta of eutherian mammals is critical for fetal development and growth during gestation. The allantois first appears at embryonic day (E) 7.5 as a bud of mesoderm that emerges from the posterior end of the primitive streak [1], [2], and then grows into the exocoelomic cavity, cavitates between E7.5 and E8.25 and undergoes chorio-allantoic fusion [3]. Formation of endothelium occurs de novo within the allantois at the headfold (HF) stage, beginning in the distal allantois with the appearance of Flk1-positive angioblasts, which are precursors of endothelial cells (ECs) [4], [5]. Specification of angioblasts and their morphogenesis into endothelial tubes (ETs) then proceeds proximally to the base of the allantois, where nascent allantoic blood vessels fuse with those of the embryo to create a continuous vasculature throughout the embryo and yolk sac [1], [5]. The allantois vessel network, also known as a vascular plexus, is ultimately remodeled into an umbilical artery, umbilical vein and the fetal vessels of the placenta. The vascular plexus of the allantois presumably promotes the growth of mural cells to provide structural support for the vascular walls, similar to the yolk sac vascular plexus [6]. Extracellular matrix (ECM) is also present, and although there have been some reports of the presence of specific matrix components in the allantois [7], [8], [9], [10], the presence of mural cells and the composition of the ECM is largely unknown.

Following chorio-allantoic fusion, the chorion forms villi into which the allantois vasculature grows, ultimately forming the labyrinthine layer of the placenta. Other components of the placenta include the outermost, maternally-derived decidual layer, the giant cell layer derived from trophectoderm and the spongiotrophoblast layer derived from polar trophectoderm. Defective development of any of these layers can lead to placental insufficiency and, in severe cases, embryonic death [11].

Mutation of the T-box transcription factor gene *Tbx4* results in abnormal vascular development in the allantois, loss of cavitation, apoptosis and lack of chorio-allantoic fusion leading to embryonic death at E10.5. Pecam, a marker of ECs, is abundantly expressed in cells of *Tbx4* mutant allantoises but these ECs do not coalesce into a primary vascular plexus [12]. Comparison of *Tbx4* RNA and Pecam protein localization as well as lineage tracing using a *Tbx4-cre* allele suggests that neither the ECs of the umbilical vessels nor their precursors express *Tbx4* [13]. In spite of this, *Tbx4* null mutants show a defect in allantois EC organization suggesting that Tbx4 plays a non-cell-autonomous role in formation of the vascular plexus.

Thus, we took a candidate gene approach to find *Tbx4* target genes expressed in the mesenchyme that could explain this non-cell-autonomous effect. Candidates were chosen if their loss either leads to chorio-allantoic fusion defects, cavitation defects or results in a vascular phenotype [14] similar to the *Tbx4* mutant allantois vascular phenotype. We analyzed expression of ECM components: *Hyaluronic acid synthase2* (*Has2*) [15], because hyaluronic acid is involved in allantois cavitation [10], [16], which is disrupted in *Tbx4* mutants [12]; *Versican* (*Vcan*) [17], which is a chondroitin sulphate proteoglycan binding partner for hyaluronic acid; *α5 integrin* (*Itgα5*) and *Fibronectin* (*Fn*), ECM genes known to be essential for development of endothelial tubes [18]. We examined components of several signaling pathways: *Bmp2*, *Bmp7* and *Bmp5*, which are implicated in chorio-allantoic fusion [19], [20]; *Wnt2*, one of the canonical Wnt family members, which is expressed in the allantois and important for placentation [21]; *Notch2*, which is expressed in the allantois [22]; *Delta like 4* (*Dll4*) and *Jagged1* (*Jag1*), ligands for Notch receptors, implicated in vascular development [23], [24]. Among transcription factors, we analyzed expression of *Twist* and *Snai1*. *Twist* has been shown to be important for *Snai1* expression [25]; epiblast-specific deletion of *Snai1* leads to a vascular phenotype in which the ECs of the embryo and allantois express Pecam but fail to coalesce to form ETs [26], similar to *Tbx4* mutant allantoises.

We found that expression of multiple ECM genes, signaling molecules and transcription factors is affected in *Tbx4* mutants, some of which have conserved T-box binding sites in their promoters. We further show that canonical Wnt signaling contributes to vessel-forming potential of the ECs of allantoises *ex vivo* and growth of the umbilical vessels into the placenta *in vivo*.

## Results

### *Tbx4* Mutant Allantoises Fail to Form a Vascular Plexus *ex vivo*

**Figure 1:**
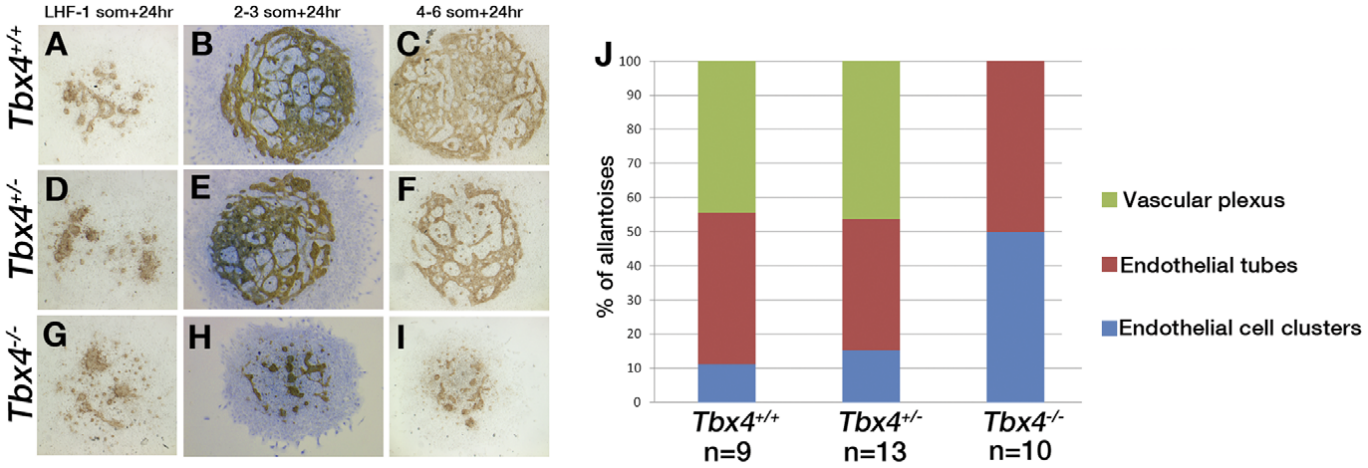
*Tbx4* mutant allantoises fail to form a vascular plexus *ex vivo*. (**A–C**) *Tbx4* ^+/+^ (**D–F**), *Tbx4^+/−^* and (**G–I**) *Tbx4^−/−^* allantoises isolated from different stage embryos were cultured for 24 hours, stained with anti-Flk1 antibody and scored for formation of isolated EC clusters, ETs or a vascular plexus. Cultures in B, E, H were counterstained with methylene blue. (**J**) Stacked bar graphs show the relative proportions of allantoises from LHF to 6 somite stage embryos of each genotype that formed EC clusters, ETs, or a vascular plexus.

To investigate their vessel-forming potential, *Tbx4* mutant and control allantoises were cultured *ex vivo* for 24 hours starting from the late head fold (LHF) stage to the 6 somite stage (E8–8.5). Wild-type or *Tbx4* heterozygous allantoises from the earliest developmental stages give rise to clusters of ECs, whereas explants from later stages spread out and give rise to a network of interconnected ETs that form a plexus [27], [28], as shown by Flk1 antibody staining (Figure 1A–F). *Tbx4* mutant allantoises, on the other hand, had clusters of ECs or ETs but failed to form a vascular plexus of interconnected ETs even in explants from the most advanced embryos (Figure 1G–I). Methylene blue nuclear staining (Figure 1B,E,H) shows the extent of allantois outgrowth. The proportion of explants of each genotype that proceeded to form EC clusters, ETs or a vascular plexus is shown in Figure 1J. Because *Tbx4* mutant allantoises show increased apoptosis [12], we tested whether decreased cell numbers affected plexus formation. When split in half, control allantoises isolated at 2–4 somites (E8.25) still formed a vascular plexus upon culture, although the plexus was smaller than that from whole allantoises (data not shown) suggesting that reduced cell number alone was not the cause of lack of plexus formation in *Tbx4* mutants. As *Tbx4* is not expressed in ECs of the allantois and cells derived from *Tbx4*-expressing cells never contribute to the endothelium of the umbilical vessels [13], these data suggest that *Tbx4* plays a non-cell-autonomous role in the development of allantois vasculature. Thus, *Tbx4* could either regulate the developing mesenchyme surrounding the ECs or the ECM through which the ECs migrate in order to form ETs and a primary plexus.

### *Tbx4* is Upstream of ECM Molecules, Signaling Molecules and Transcription Factors

**Figure 2:**
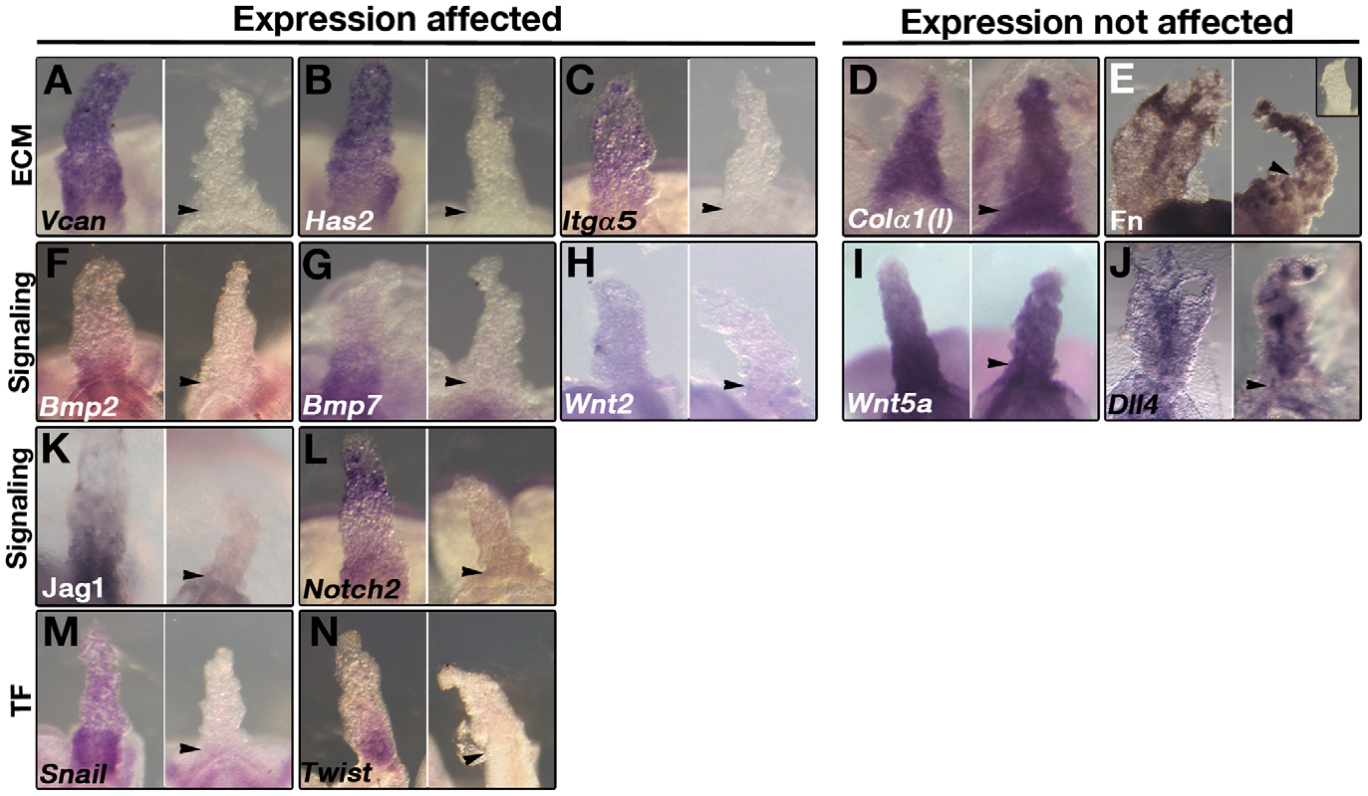
Identification of genes downstream of *Tbx4* in allantois using a candidate gene approach and ISH or IHC. In each panel the image on the left is a control and on the right is a stage-matched *Tbx4* mutant allantois. (**A–E**) Expression of ECM genes *Vcan*, *Has2* and *Itgα5* was undetectable whereas *Colα1(I)* and Fn were expressed in *Tbx4* mutants. Inset in E shows secondary antibody control in the absence of primary antibody against Fn. (**F–L**) Expression of signaling molecules *Bmp2*, *Bmp7*, *Wnt2*, Jag1, and *Notch2* was undetectable; *Wnt5a* was unaffected; *Dll4* was expressed in an EC-like pattern in the *Tbx4* mutants. (**M–N**) Expression of transcription factors (TF) *Snai1* and *Twist* was undetectable in the *Tbx4* mutant allantoises. The allantoises are shown in ventral views except the mutant allantois in panel (H) and the control and mutant allantois in panel (N) which are side views.

To explore the role of *Tbx4* in the development of the allantois we analyzed the expression of various ECM markers, signaling molecules and transcription factors in a candidate gene approach. Components of the ECM *Vcan* and *Has2* were expressed in wild-type allantoises but were undetectable in *Tbx4* mutant allantoises (Figure 2A,B). Expression of *Itgα5* was undetectable (Figure 2C) whereas Fn and *Collagen αI (1)* (*Colα1(I)*) were expressed in *Tbx4* mutants (Figure 2D,E).

**Figure 3:**
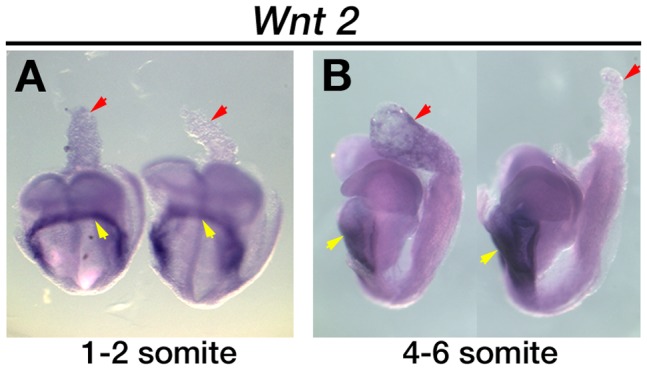
*Wnt2* expression in *Tbx4* mutant embryos. (**A,B**) Expression of *Wnt2* in *Tbx4* mutant embryos at 1–2 somite (A) and 4–6 somite (B) stages. Embryos on the left are controls and on the right are stage-matched *Tbx4* mutants. In *Tbx4* mutant allantoises, expression was not detected above background levels. Red arrowheads, allantois; yellow arrowheads, heart.

Expression of both *Bmp2* and *Bmp7* was undetectable in *Tbx4* mutant allantoises (Figure 2F,G). *Wnt2* is expressed throughout the process of growth and vascularization of the allantois starting at the LHF stage [21]. *Wnt2* expression was undetectable in *Tbx4* mutant allantoises (Figure 2H and Figure 3). On the other hand, non-canonical *Wnt5a* was expressed in mutant allantoises (Figure 2I). *Dll4* was expressed in *Tbx4* mutant allantoises in EC clusters, which fail to form vessels (Figure 2J). *Jag1* and *Notch2* were expressed in control allantoises but were not detected in *Tbx4* mutant allantoises (Figure 2K,L). Similarly, *Snai1* and *Twist* were not detected in *Tbx4* mutant allantoises (Figure 2M,N).

### Agonists of the Canonical Wnt Signaling Pathway Rescue and a Wnt Signaling Antagonist Phenocopies the *Tbx4* Mutant Phenotype *ex vivo*

**Figure 4:**
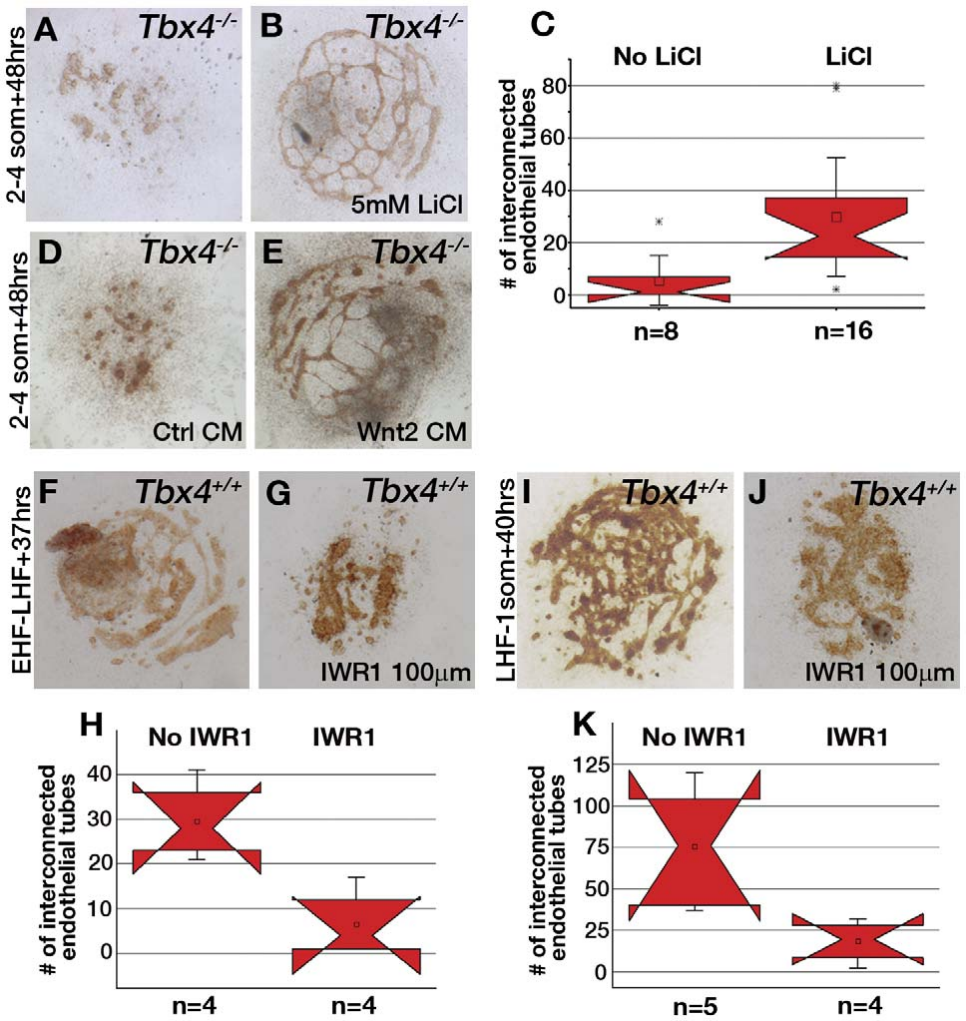
Wnt signaling is essential for vessel formation in allantoises *ex vivo*. (**A,B**) Vascular plexus formation is rescued in *Tbx4* mutant allantoises in the presence of 5 mM LiCl. (**C**) Quantification of the number of ET interconnections in the absence and presence of LiCl using notched box plots (bars represent 1.5 standard deviations and outliers are indicated by *). When the notches do not overlap, the medians are significantly different at ∼95% confidence level. (**D,E**) Wnt2 conditioned media but not control conditioned media rescues plexus formation in mutant allantoises. (**F–K**) IWR1 inhibits plexus formation in control allantoises explanted at EHF-LHF (F,G,H), or LHF-1 somite stage (I,J,K). Notched box plots (H,K) show significantly lower number of interconnections in the presence of IWR1 (bars represent ranges). Cultures were stained with anti-Flk1 antibody.

Because canonical *Wnt2* was not expressed above background in *Tbx4* mutant allantoises (red arrows Figure 3A,B) although it was expressed in hearts of *Tbx4* mutant embryos (yellow arrows Figure 3A,B), we examined the contribution of the canonical Wnt signaling pathway to the vessel-forming potential of the allantois under the control of *Tbx4*. The canonical Wnt signaling pathway was activated in culture using LiCl, an inhibitor of GSK3-β and thus an activator of β-catenin-mediated transcription [29]. When treated with LiCl, *Tbx4* mutant allantois explants formed ETs which interconnected and formed a vascular plexus *ex vivo* (Figure 4B) unlike mutant allantoises cultured in the absence of LiCl, which never formed a vascular plexus (Figure 4A). The total number of interconnections of the ETs in the vascular network was compared in rescued and control mutant allantoises as a measure of the extent of plexus formation (Figure 4C) and was shown by notched box plots to be significantly different between control and LiCl treated mutants [30] (Mann Whitney U test p = 0.0016). Similar rescue of ET and plexus formation was seen when *Wnt2* conditioned media was added to mutant allantois cultures (control mutants, n = 2, number of interconnections 1, 10; mutants with Wnt2 conditioned media, n = 2, number of interconnections 54, 63) (Figure 4D, E).

IWR1, a small molecule inhibitor, was used to block the canonical Wnt signaling pathway at a concentration found to be non-toxic to cells in organ culture [31]. Allantoises from wildtype embryos were isolated at EHF-LHF (Figure 4F,G,H) and LHF-1 somite stages (Figure 4I,J,K) and IWR1 in DMSO or DMSO vehicle alone was added to the cultures. In the presence of inhibitor, wildtype allantoises failed to form a vascular plexus but showed formation of EC clusters positive for Flk1. Taken together, these results suggest that canonical Wnt signaling contributes to the vessel-forming ability of allantoises *ex vivo*, and in the absence of this signal the ECs fail to form a primary vascular plexus.

### *Tbx4* and *Wnt2* Genetically Interact during the Formation of the Chorio-allantoic Placenta

Since canonical Wnt signaling is necessary for formation of allantois vessels *ex vivo* we tested whether *Tbx4*-mediated control of canonical Wnt signaling alone was responsible for vessel formation in allantoises *in vivo*. *Wnt2* is the only canonical Wnt known to be expressed in the allantois and thus we analyzed *Tbx4^+/−^*;*Wnt2^+/−^* and *Tbx4^+/−^;Wnt2^−/−^* allantoises. Allantoises from both these genotypes fused with the chorion normally at E8.5 and formed normal umbilical vessels at E10.5 (data not shown) indicating no genetic interactions between *Tbx4* and *Wnt2* during allantois vessel formation *in vivo*. We hypothesized that lack of a phenotype in the *Tbx4^+/−^;Wnt2^−/−^* allantoises could be due to activation of *β-catenin* by another pathway. Thus, in order to create a mesenchymal deletion of *β-catenin*, we used a *Tbx4-cre* [13] and a *β-catenin* conditional allele [32]. *Tbx4^cre/+^; βcatenin^cond/cond^* embryos did not show an allantoic vascular phenotype (data not shown).

**Figure 5:**
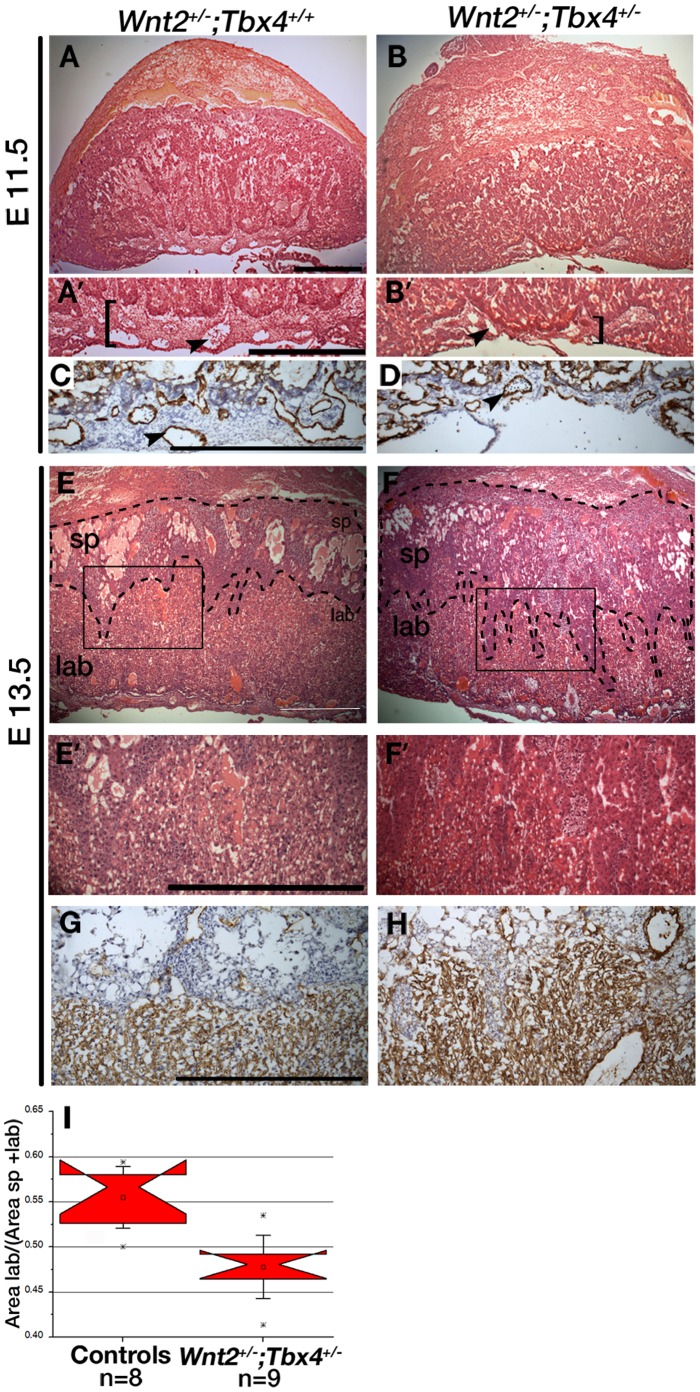
Genetic interactions between *Tbx4* and *Wnt2* in formation of the chorio-allantoic placenta. (**A–B’**) Morphology of control and *Tbx4^+/−^*;*Wnt2^+/−^* placentas at E11.5. A’ and B’ are higher magnification views of the chorio-allantoic plate, the width of which is indicated by the brackets. (**C,D**) Pecam staining of chorio-allantoic plate at E11.5. Arrowheads point to vessels. (**E–F’**) At E13.5, increased interdigitation of spongiotrophoblast (demarcated by dashed lines) and labyrinthine layers is observed in double heterozygote placentas. E’ and F’ are higher magnification views of boxed regions in E and F. (**G,H**) Pecam staining of E13.5 placentas. (**I**) Notched box plots show a significantly lower ratio of area of labyrinth/(area of spongiotrophoblast + labyrinth) in the double heterozygotes compared with controls at E13.5 (bars represent 1.5 standard deviations and outliers are indicated by *). sp, spongiotrophoblast, lab, labyrinthine layer. Scale bars: 500 µm.

To determine if *Tbx4* and *Wnt2* interact later during allantois-derived vessel formation, chorio-allantoic placenta development of *Tbx4^+/−^*;*Wnt2^+/−^* embryos was analyzed. At E11.5, there is a decreased amount of perivascular stroma in the chorio-allantoic plate of double heterozygous placentas (Figure 5A–D). The extent of vascularization of the chorio-allantoic plate was analyzed using H & E sections of controls and double heterozygote placentas (Figure 5A,B). Quantification of the vessels shows a significantly lower number of vessels present in double heterozygotes compared to controls (Mann Whitney U test, p = 0.001). Identification of vessels was confirmed by Pecam staining (Fig. 5C,D). At E13.5, double heterozygotes showed increased interdigitation of the spongiotrophoblast and labyrinth (Figure 5E,F,G,H). Additionally, at E13.5 there was a significant reduction in the labyrinthine layer as seen from the ratio of area of labyrinth to the combined area of labyrinth and spongiotrophoblast in double heterozygotes (Figure 5I, Mann Whitney U test, p = 0.002). These results indicate that *Tbx4* and *Wnt2* interact to form the allantois-derived vasculature of the chorio-allantoic plate and the labyrinthine layer of the placenta.

### Genes Affected in *Tbx4* Mutant Allantoises have Conserved T-box Binding Sites

**Figure 6:**
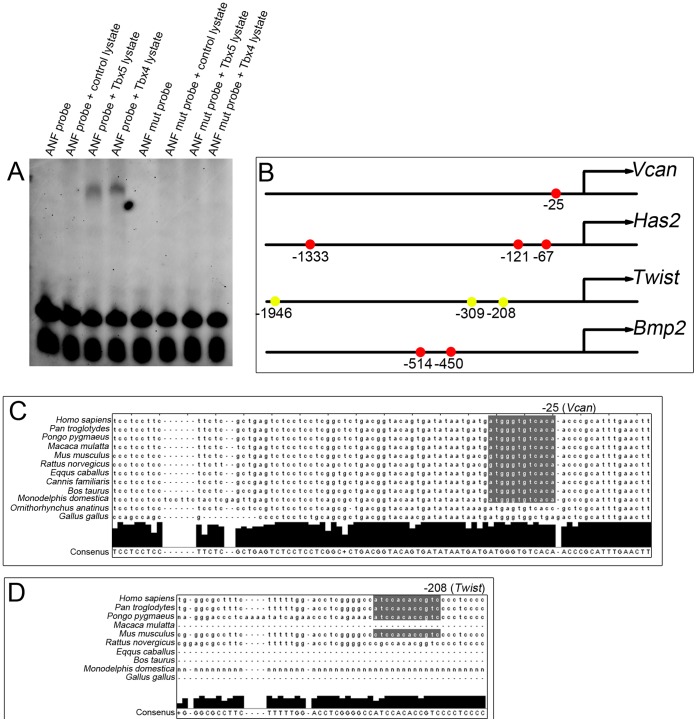
*Vcan*, *Has2*, *Twist* and *Bmp2* contain conserved T-box binding sites. (**A**) EMSA showing binding of TBX5- and Tbx4-containing cell lysates to wildtype T-box binding site-containing *ANF* probe but not the mutant *ANF* probe. (**B**) Conserved T-box binding sites present in the promoter regions of *Vcan*, *Has2*, *Twist* and *Bmp2* genes. Yellow circles highlight conservation between at least human and mouse; red circles highlight conservation between >5 mammalian species including human and mouse. Numbers refer to human sequences as per ConTra. (**C**) Highly conserved site (gray shading) at the −25 position in the promoter of the *Vcan* gene. (**D**) Conserved site (gray shading) at the −208 position in the promoter of the *Twist* gene.

Tbx4 and its closely related paralogue Tbx5 share 94% amino acid identity in their T-box domains [33]. Although the DNA binding sequence for Tbx4 has not been characterized, we tested whether Tbx4 protein is capable of binding to the TBX5 consensus T-box binding element (TBE) in the *ANF* promoter [34]. When incubated with cell lysates containing either mouse Tbx4 or human TBX5, probe containing the *ANF* TBE showed a mobility shift which was lost when the core sequence of the TBE was mutated (Figure 6A) confirming that Tbx4 can bind the TBX5 TBE. We then used ConTra [35] to examine 2 Kb of the promoter region of the genes affected in Tbx4 mutant allantoises for this element and, where it was present, analyzed its conservation. Four out of the ten genes affected in the *Tbx4* mutant allantoises – *Vcan*, *Has2*, *Twist* and *Bmp2*– have TBE’s conserved in at least mouse and human (Figure 6B, yellow circles) or more than 5 mammalian species including mouse and human (Figure 6B, red circles). An example of site conservation between multiple mammalian species including mouse and human upstream of *Vcan* is shown in Figure 6C and an example of site conservation between human and mouse upstream of *Twist* is shown in Figure 6D.

## Discussion

*Tbx4* affects multiple processes important for allantois development including chorio-allantoic fusion, cavitation, cell proliferation, apoptosis and vasculogenesis [12]. Here we show evidence that *Tbx4* plays a role in these processes by regulating expression of a variety of ECM genes, signaling molecules and transcription factor genes either directly or indirectly. We show that expression of ECM molecules *Vcan, Has2*, and *Itgα5*, transcription factors *Snai1* and *Twist*, and signaling molecules *Bmp2*, *Bmp7*, *Notch2*, *Jag1* and *Wnt2* is undetectable in *Tbx4* mutant allantoises. Further, we show that Tbx4 could potentially directly regulate the expression of *Vcan*, *Has2*, *Twist* and *Bmp2*, as they have conserved T-box binding sites in their promoters. Additionally, individual mutations in some of these genes show that their absence alone would be enough to explain the EC phenotype of *Tbx4* mutant allantoises. For example, an epiblast-specific mutation in *Snai1*, which is absent from *Tbx4* mutant allantoises, results in the lack of a vascular plexus in the embryo proper and in allantois explants, although explants show the presence of differentiated Pecam-positive ECs [26]. *Itgα5* [18] and *Wnt2* mutant embryoid bodies [36] that are differentiated along the vascular pathway show Pecam-positive ECs but do not form a vascular plexus; neither of these genes is detected in *Tbx4* mutant allantoises. The cell adhesion molecule *Vcam1* is lacking in *Tbx4* mutant allantoises, which potentially explains their lack of chorio-allantoic fusion [12].

Four of the genes absent in *Tbx4* mutant allantoises, *Vcan*, *Jag1*, *Snai1* and *Twist,* are downstream targets of the canonical Wnt signaling pathway in different systems (http://www.stanford.edu/group/nusselab/cgi-bin/Wnt/target_genes), indicating a role for canonical Wnt signaling in development of allantois vasculature downstream of *Tbx4*. Additionally, since *Tbx4* is not expressed in ECs of the allantois but its absence still causes a drastic effect on the assembly of ECs into a vascular plexus, loss of a secreted molecule like *Wnt2* could explain this non-cell-autonomous effect. There is evidence that *Wnt2* plays an important role in EC proliferation and network formation specifically in hepatic sinusoidal ECs (HSEC’s). Wnt inhibitors lead to reduced proliferation and reduced endothelial-tube-forming ability of HSEC’s on matrigel [37]. Furthermore, *Wnt2* null embryoid bodies, which fail to form a Pecam-positive vascular plexus, can be rescued by the addition of Wnt2 conditioned media [36]. We were able to rescue the vessel-forming ability of *Tbx4* mutant allantoises by activating the Wnt signaling pathway using LiCl or by culturing in Wnt2 conditioned media. We were also able to disrupt vascular plexus formation in wildtype allantoises by addition of a tankyrase inhibitor IWR1. Tankyrase destabilizes axin and prevents β-catenin from degradation, thus in the presence of the inhibitor, β-catenin is degraded, blocking the canonical Wnt signaling pathway [31]. Taken together, our results suggest for the first time that canonical Wnt signaling contributes to the vessel-forming ability of allantoises *ex vivo*, and in the absence of this signal the ECs fail to form a primary vascular plexus.

*Tbx4* and *Wnt2* do not show a genetic interaction during allantoic vessel formation *in vivo*. One possible explanation is that other, as yet unknown, canonical Wnts are expressed in the allantois. Alternatively, β-catenin could be activated downstream of *Tbx4* by other pathways that are still active in *Tbx4^+/−^*;*Wnt2^+/−^* and *Tbx4^+/−^;Wnt2^−/−^* allantoises. It has already been shown that conditional excision of *β-catenin* in ECs does not affect allantois vascular development [38]. Thus, neither an endothelial [38] nor a mesenchymal deletion of *β-catenin* (our study) reproduces the *Tbx4* mutant phenotype, suggesting other pathways downstream of Tbx4 are involved. The presence of conserved TBEs in the promoters of *Vcan*, *Has2*, *Twist* and *Bmp2* make these genes good candidates for direct regulation by Tbx4 during the development of the allantois vasculature.

It is also possible that *Tbx4* and *Wnt2* may interact in formation of allantois vasculature but a phenotype is not evident until later in development in the allantois-derived placental vasculature. This is the case with the HoxA genes – *HoxA10*, *HoxA11* and *HoxA13*– all of which are expressed in the allantois. The expression of the HoxA genes is essential during a brief window of allantois development but the mutant phenotype is only evident at later stages as a disruption of placental vasculature [39]. Thus, it may be a common theme for some genes expressed in the allantois to manifest a phenotype in the placenta by regulating the development of the allantois. We show that indeed, *Tbx4* and *Wnt2* interact in double heterozygotes in the formation of placental vasculature. By itself *Wnt2* is important for the proper formation of the different placental layers and *Wnt2* null mutants show disruptions in the placental labyrinthine layer [21]. In addition, canonical Wnt signaling has been shown to be important for placenta development; deficiency in a number of genes involved in Wnt/β-catenin signaling show mid-gestation lethality due to placental insufficiency. For example, conditional inactivation of *β-catenin* in ECs shows a phenotype similar to *Wnt2*;*Tbx4* double heterozygotes, where the labyrinthine layer is smaller and less vascularized [38]. *Rspondin3*, a secretory molecule whose proposed function is to promote the canonical Wnt signaling pathway, is expressed in the allantois. Fetal vessels of *Rspondin3* null embryos fail to penetrate the chorion leading to lethality during mid-gestation [40]. Similarly, the Wnt receptor *Fzd5* is expressed in the labyrinthine layer and null mutants for *Fzd5* die due to lack of penetration of fetal vessels into the chorion [41]. We show that the *Wnt2*;*Tbx4* double heterozgygous placentas have decreased vascular coverage in the chorioallantoic plate, again suggesting the importance of *Wnt2* in the vessel-forming potential of ECs derived from the allantois. Furthermore, double heterozygous placentas show increased interdigitation of placental layers and reductions in the labyrinthine layer of the placenta. Thus, although *Wnt2* does not genetically interact with *Tbx4* in the process of vessel formation in the allantois, these genes do interact in formation of the vasculature of the chorio-allantoic plate and the labyrinthine layer of the placenta.

## Materials and Methods

### Mice

Mice carrying a *Tbx4* null allele, *Tbx4^tm1^.^2Pa^* [12], hereafter referred to as *Tbx4^−^*, a *Wnt2* null allele, *Wnt2^tm1^.^1(rtTA)Eem^* [42], hereafter referred to as *Wnt2^−^*, a *Tbx4-cre* allele, an insertion into the endogenous *Tbx4* locus resulting in a bicistronic allele that expresses both *cre* and *Tbx4* and has been shown to be expressed in all areas of *Tbx4* expression [13] and a *β-catenin* loss-of-function conditional allele [32], hereafter referred to as *βcatenin^cond^*, were genotyped as described previously.

### Histology and Vessel Counts in the Chorio-allantoic Plate

Embryos and placentas were collected from timed matings and dissected out of the decidua; yolk sacs were removed for genotyping and the placentas were fixed in Bouin’s fixative (Sigma). After dehydration in ethanol, placentas were embedded in paraffin wax, sectioned at 10 µm and stained with hematoxylin and eosin (H & E). From cross sections through the center of the E11.5 placenta, every 5^th^ section (for a total of 15–20 sections/placenta) was used to quantitate vasculature by counting the number of vessels per section present in the chorio-allantoic plate. The total number of vessels per placenta was used as a representation of the amount of vasculature in the chorio-allantoic plate. Three to five placentas were analyzed for each genotype. At E13.5, quantitation of the area of labyrinthine and spongiotrophoblast layers of placenta was done on H & E stained sections using the software NIS Elements (Nikon). For antibody staining, placentas were fixed in paraformaldehyde, equilibrated in 30% sucrose, embedded in OCT (Tissue-Tek) and cryosectioned at 10 µm.

### *In situ* Hybridization and Immunohistochemistry

*In situ* hybridization (ISH) and immunohistochemistry (IHC) were performed according to standard protocols [43], [44]. ISH with sense RNA controls showed no background staining. Primary antibodies used include anti-Flk1 (R&D), anti-Jag1 (R&D), anti-Fibronectin (SCBT) and anti Pecam (BD Biosciences); secondary antibodies were peroxidase-conjugated donkey IgG (Jackson Immunochemicals). Secondary antibody controls showed no background staining. For each ISH or IHC, 2 to 7 mutant embryos (E8.25–8.5; 1–6 somites) were analyzed.

### *Ex vivo* Allantois Culture

Allantoises were aspirated using glass pipettes to obtain the full length from distal tip to the site of attachment to the amnion and yolk sac [28] and transferred to 24 well dishes containing 0.5 ml of rat serum (Pel-Freeze Biologicals) and DMEM (Invitrogen) in a 1∶1 ratio. The allantoises were scored for developmental progression based on formation of EC clusters, ETs or a vascular plexus at the end of 24 hours. For rescue experiments, lithium chloride (LiCl) was added to the media at a final concentration of 5 mM. Wnt2 producing CHO cells were used to make Wnt2 conditioned media and vector transfected CHO cells were used to make control conditioned media [37]. For conditioned media rescue experiments, allantoises were cultured in 50% rat serum, 25% control or Wnt2-conditioned media and 25% DMEM. For inhibition of canonical Wnt signaling, 100 µM IWR1 (Cayman Chemicals) prepared in DMSO, a concentration shown to be non-toxic to cultured cells [31] or DMSO alone was added to cultures and allantoises were cultured for 36–40 hours. For antibody staining, the cultures were fixed overnight in 4% PFA, washed in PBT, and treated for IHC for Flk1. For each experiment n was ≥2.

### Generation of Fluorescent Probes Containing TBE Upstream of *ANF*

To generate probe, 12 bp linker sequence (*acg agt ctc tac*) was added to the 3′ end of the reverse complement of the TBX5 TBE in the promoter region upstream of the ANF start site (−275 to −246) to generate a wildtype T-box binding sequence, 5′gct ccc act tca aag gtg tga gaa gag taa *acg agt ctc tac* 3′. To generate a mutant version of this site the core sequence of the T-box binding element was mutated to get the sequence 5′gct ccc act tca aag gga tga gaa gag taa *acg agt ctc tac* 3′ (the residues mutated have been underlined and the linker is italicized). A fluorescently labeled oligonucleotide complimentary to the linker sequence, Cy5- 5′ *gta gag act cgt* 3′, was annealed to both these sequences and filled using Klenow polymerase to generate the fluorescently labeled ANF wildtype and mutant double stranded probes, which were used for the electrophoretic mobility shift assay (EMSA).

### Preparation of Cell Lysates Containing Tbx4 and Tbx5 Protein and EMSA

To generate Tbx4 and Tbx5 protein, reactions were carried out using a transcription translation coupled reticulolysate system (TNT® T7 Quick Coupled Transcription/Translation System, Promega) according to the manufacturer’s protocol. 1ug of human *TBX5* cDNA [34] and mouse *Tbx4* cDNA [45] was incubated in a 50 µl reaction, for 1 hour at 30°C.

Cell lysates containing Tbx4 or Tbx5 protein were incubated with approximately 0.3 ng of fluorescently labeled probe in binding buffer (20 mM HEPES, pH 7.5, 50 mM KCl, 5 mM MgCl_2_, 10 µM ZnCl_2_, 6% glycerol, 200 µg of bovine serum albumin per ml, and 50 µg of poly(dI-dC)·poly(dI-dC) per ml [46]) for 20 minutes at room temperature. This reaction was then loaded onto a 4% polyacrylamide gel, samples were run for 3 h at 120 V at room temperature, the gel was vacuum dried and imaged using Typhoon TRIO variable mode imager (Amersham Biosciences). Probe alone or probe with empty lysates (without cDNA) were used as negative controls.
